# *In vitro*–*In vivo* efficacy of *Trichoderma asperellum* and *Trichoderma virens* against *Ganoderma boninense* in oil palm seedlings

**DOI:** 10.1080/15592324.2026.2671575

**Published:** 2026-05-13

**Authors:** Puguh Prastiyo Hutomo, Rizqika Yanuar Maghfiro, Abdul Latief Abadi, Aziz Natawijaya, Teruo Sone, Adi Setiawan, Muhammad Akhid Syib’li

**Affiliations:** a Graduate Plant Pathology Study Program, Faculty of Agriculture, Universitas Brawijaya, Malang, Indonesia; b Department of Plant Pest and Disease, Faculty of Agriculture, Universitas Brawijaya, Malang, Indonesia; c Department of Corporate Development, PT. Bumitama Gunajaya Agro, Jakarta, Indonesia; d Research Faculty of Agriculture, Hokkaido University, Sapporo, Japan; e Department of Agronomy, Faculty of Agriculture, Universitas Brawijaya, Malang, Indonesia

**Keywords:** Basal stem rot, *Ganoderma*, oil palm seedlings, *Trichoderma asperellum*, *Trichoderma virens*, dual culture assay, greenhouse bioassay

## Abstract

Oil palm (*Elaeis guineensis* Jacq.) is a strategic commodity in tropical regions, serving as a major source of vegetable oil. However, its productivity is threatened by basal stem rot (BSR) associated with *Ganoderma* spp., which can cause severe yield losses. This study evaluated the biocontrol potential of *Trichoderma asperellum* and *Trichoderma virens* against a pathogenic *Ganoderma* isolate using morphological and molecular identification, *in vitro* dual-culture assays, and greenhouse bioassays. Molecular analysis indicated that isolate UBGb.1 was closely related to *G. boninense* (99.88% similarity, based on Tef1α sequencing), while UBPg.3 and EXPg.2 were identified as *T. asperellum* and *T. virens* (100% similarity), respectively. *In vitro*, *T. asperellum* inhibited *Ganoderma* mycelial growth by 51.14%, outperforming *T. virens* (35.90%). *In vivo* assays showed that increasing pathogen inoculum dose increased disease development in oil palm seedlings. Under beneficial microbe application, *T. asperellum* (140 g pot⁻¹) suppressed visible disease symptoms for 42 d after inoculation (DAI) (DSI = 0%), whereas the consortium treatment resulted in higher disease severity than the single-strain treatment (DSI 10.88% at 42 DAI). Overall, *T. asperellum* consistently showed superior performance compared with *T. virens*. These results suggest that nursery-scale biocontrol efficacy depends on strain stability and consistent antifungal activity, and that incompatibility between strains may reduce performance due to physiological competition.

## 1. Introduction

Oil palm (*Elaeis guineensis* Jacq.) is a strategic commodity in tropical regions and a major source of vegetable oil. Despite its economic importance, oil palm productivity is severely constrained by basal stem rot (BSR), a destructive disease commonly associated with *Ganoderma boninense*. The pathogen is soil-borne and can colonize the root and basal stem tissues, disrupting water and nutrient transport and leading to root necrosis, foliar yellowing, frond collapse, and eventual palm death. BSR has become one of the most serious threats to the palm oil industry in Southeast Asia, with severe infestations reported to cause substantial yield losses and significant economic impacts.^1^ Within Southeast Asia, BSR has been documented for decades. In Indonesia, the disease has been reported since the 1980s, including outbreaks in North Sumatra (Adolina, Gunung Bayu, and Tinjowan).^2^ In Malaysia, disease escalation in infected blocks can drive long-term productivity decline and increased replanting costs, highlighting the need for practical and scalable management options.^3^

Managing BSR remains challenging because the pathogen persists in soil and infected residues, spreads through root contact, and may remain asymptomatic during early infection. Once external symptoms become visible, internal colonization is often extensive and difficult to reverse, reducing the effectiveness of reactive control measures. The pathogen can also survive in infected tissues and form structures that tolerate adverse environmental conditions, contributing to long-term inoculum carryover in plantations.^4^ As a result, strategies that can be implemented earlier, particularly at the nursery stage, are increasingly considered critical to reducing initial infection pressure and improving field establishment of healthy planting materials.

Various approaches have been explored to suppress BSR, including sanitation, removal of infected palms and residues, soil and nutrient management, and chemical fungicides. However, chemical control may show limited field consistency for soil-borne pathogens and can raise concerns related to environmental safety and long-term sustainability. Biological control offers an alternative pathway, especially using antagonistic fungi such as *Trichoderma* spp., which are widely recognized for their ability to colonize the rhizosphere, compete for space and nutrients, produce antifungal metabolites, and directly inhibit pathogens through mycoparasitism and lytic enzymes. Importantly, the efficacy of *Trichoderma* is often strain-dependent and may vary across experimental systems (*in vitro* versus greenhouse) and across application modes. In addition, while multi-strain consortia are sometimes proposed to broaden functional traits, strain incompatibility or competition may reduce overall performance compared with the best single strain.

Therefore, this study aimed to (i) identify the pathogenic *Ganoderma* isolate and two antagonistic *Trichoderma* isolates using morphological and molecular approaches, (ii) quantify *in vitro* antagonism under dual-culture assays, (iii) characterize disease development under different pathogen inoculum doses in oil palm seedlings, and (iv) evaluate greenhouse efficacy of single-strain versus consortium applications in suppressing BSR severity. We hypothesized that *Trichoderma* treatments would reduce disease severity *in vivo*, while consortium performance may not necessarily exceed the best single strain due to potential inter-strain competition and niche overlap. To address these objectives, we conducted a stepwise evaluation beginning with isolate identification and *in vitro* antagonism screening, followed by dose-response assessment of pathogen inoculum, and finally greenhouse validation of selected *Trichoderma* treatments on oil palm seedlings. This workflow was designed to connect laboratory evidence with practical performance at the seedling stage, where early intervention is expected to provide the greatest benefit for subsequent field establishment.

## 2. Materials and methods

### 2.1. Fungal isolates

*Ganoderma boninense* (isolate UBGb.1), *Trichoderma asperellum* (isolate UBPg.3), and *Trichoderma virens* (isolate EXPg.2) were used in this study. *G. boninense* and *T. asperellum* were obtained from the Plant Disease Laboratory, Department of Plant Pest and Disease, Faculty of Agriculture, Universitas Brawijaya. In contrast, *T. virens* was isolated in this study from root and seed tissues of diseased oil palm seedlings collected from a nursery. Molecular identification of both *Trichoderma* isolates was carried out in this study, as detailed in Section 2.4. For isolation, plant tissues were surface-sterilized using 70% ethanol for 30 s, 1% NaOCl for 1 min, followed by three rinses with sterile distilled water, blotted dry, and placed on potato dextrose agar (PDA). All isolates were purified by hyphal-tip subculture on fresh PDA under aseptic conditions and incubated at 25–27 °C for 7 d until vigorous mycelial growth was observed.

### 2.2. Planting medium preparation and seedlings

The planting medium used was sterile soil. The seeds were obtained from the Dumpy Sungai Pancur variety, imported from the Center for Oil Palm Research, Medan, Indonesia. The oil palm seeds were previously soaked in water for one hour to aid dormancy breaking. After soaking, the seeds were drained on a dry tissue before being sown in the planting medium.

Sowing was carried out by planting oil palm seeds in 10 cm x 15 cm polybags in the greenhouse of the Faculty of Agriculture, Brawijaya University. Once all the polybags were filled with oil palm seeds, they were watered with clean water. The polybags with the seeds were placed in the greenhouse, maintaining a temperature of 23–33 °C. The seeds were watered daily and observed until the oil palm seedlings germinated. Observations were made daily to monitor the growing medium and seedling growth. The selected seeds were healthy, fresh, 60-day-old seedlings, which were transplanted into transparent pots measuring 25 cm x 25 cm.

### 2.3. Morphological characterization of fungal isolates

Morphological identification was conducted using macroscopic and microscopic observations. For macroscopic characterization, each isolate was cultured on potato dextrose agar (PDA) and incubated at 25–27 °C for 7–10 d. Colony characteristics, including color, texture, margin, and overall growth pattern, were recorded.

For microscopic characterization, *Trichoderma* isolates were examined under a compound microscope (Olympus BX41) equipped with an Olympus DP26 digital camera to document hyphal features, conidiophores, phialides, and conidia. For *Ganoderma*, microscopic observations focused on hyphal characteristics and spore morphology obtained from fruiting body/basidiocarp structures produced on the corn–sawdust–based medium. Selected samples were further examined using scanning electron microscopy (SEM: Fei Type Inspect S50) at the Advanced Minerals & Materials Laboratory, State University of Malang. SEM observations were conducted for all isolates using actively growing mycelia collected from PDA cultures. Small sample blocks (approximately 1 × 1 cm) containing actively growing mycelia were prepared, dehydrated using critical point drying, sputter-coated with a conductive metal layer, and observed under SEM following Sonmez et al.^5^

### 2.4. Molecular identification

Genomic DNA was extracted from fresh mycelia using the Quick-DNA Fungal/Bacterial Miniprep Kit (Zymo Research, D6005) according to the manufacturer’s instructions. For *Trichoderma* samples, the internal transcribed spacer (ITS) region was amplified using primers ITS1 (5′-TCC GTA GGT GAA CCT GCG G-3′) and ITS4 (5′-TCC TCC GCT TAT TGA TAT GC-3′). PCR amplification was performed using MyTaq HS Red Mix 2 × (Bioline, BIO-25048) in a total reaction volume of 25 µL containing 1 µL DNA template (~10–50 ng) under the following cycling conditions: initial denaturation at 95 °C for 3 min; 35 cycles of denaturation at 95 °C for 15 s, annealing at 52 °C for 30 s, and extension at 72 °C for 45 s; followed by a final extension at 72 °C for 3 min.

*Ganoderma* genomic DNA samples were extracted from fresh mycelia using the Quick-DNA Magbead Plus Kit (Zymo Research, D4082) according to the manufacturer's instructions. The translation elongation factor 1-alpha (Tef1a) region was amplified using primers Tef1α EF-Gano23F (5’-GGTGTCAGGCAGCTCATYGT-3’) and Tef1α EF-Gano887R (5’-CGAACTTGCARGCGATGTG-3’). PCR amplification was performed using Taq DNA Polymerase (Thermo Scientific, EP0402) in a total reaction volume of 25 µL containing 1 µL of DNA template (~10–50 ng) with the following cycling conditions: initial denaturation at 95 °C for 1 min; 35 cycles of denaturation at 95 °C for 15 s, stepped (touchdown) annealing at 62 °C, 59 °C, 56 °C, and 53 °C (15 s per step) to enhance primer specificity, and elongation at 72 °C for 30 s; followed by a final extension at 72 °C for 7 min.

PCR products were verified by agarose gel electrophoresis on 0.8% agarose gel prepared in TBE buffer and visualized under UV illumination. Amplicons (approximately 500–850 bp) were subjected to Sanger sequencing. Consensus sequences were assembled and compared against the NCBI GenBank database using BLASTn.

Closely related reference sequences were retrieved from GenBank and aligned using the Alignment Tool in MEGA 12. Phylogenetic relationships were inferred using the neighbor-joining (NJ) method with 1,000 bootstrap replicates, and evolutionary distances were computed under the Kimura 2-parameter model. For rooting purposes, the Tef1α tree (UBGb.1) and the ITS rDNA trees (UBPg.3 and EXPg.2) were rooted using sequences of the same locus from the following non-target outgroup taxa: *Trametes hirsuta* (KX880928.1), *Hypomyces lactifluorum* (PX634620.1), and *Clonostachys rosea* (MT605141.1).

### 2.5. Preparation of *Ganoderma boninense* inoculum

*Ganoderma boninense* was subcultured onto fresh potato dextrose agar (PDA) to obtain an actively growing culture, then propagated on a sterile ground corn and sawdust solid substrate under aseptic conditions following standard procedures used in our laboratory. The inoculated substrate was incubated under controlled conditions (25–27 °C, dark) until uniform mycelial colonization was achieved, and then used as the inoculum source for subsequent experiments. Inoculum preparation and application were standardized across all treatments.

### 2.6. Experiment 1: in vitro antagonistic assay

The antagonistic activity of *Trichoderma* isolates against *Ganoderma boninense* was evaluated using a dual-culture assay on potato dextrose agar (PDA). Mycelial plugs (approximately 5 mm in diameter) of *G. boninense* and each *Trichoderma* isolate were placed on the same PDA plate 3 cm apart. Plates were incubated at 25–27 °C, and colony radial growth was recorded at 1, 3, 6, 9, and 12 d after incubation. The percentage inhibition of pathogen growth was calculated using the following equation:

(1) $$I(\%)=\frac{(r_{1}-r_{2})\;}{r_{1}}\times 100\%$$

where *I* is the percentage inhibition (%), r₁ is the radius of the pathogen colony measured away from the antagonist (from the center of the pathogen plug), and r₂ is the radius of the pathogen colony measured toward the antagonist (from the center of the pathogen plug). Each treatment was performed with four independent replicates.

### 2.7. Experiment 2: *Ganoderma boninense* dosage assay on two-month-old oil palm seedlings

A dosage (dose–response) assay was conducted to evaluate disease development in oil palm seedlings under different amounts of *Ganoderma boninense* inoculum. Two-month-old seedlings were assigned to five inoculum treatments (healthy control [0 g pot^−1^], 30, 50, 70, and 150 g pot^−1^) with six replicates per treatment (Table 1). For inoculation, the required amount of colonized *G. boninense* substrate was placed evenly around the root zone until it contacted the roots and was then covered with sterile soil medium. After inoculation, seedlings were maintained under controlled conditions and monitored daily for symptom appearance and progression. Observations included disease severity (DSI), incubation period, and seedling vegetative growth parameters. Disease severity (DSI) was assessed at 7, 14, 21, and 28 DAI using the same leaf-based scoring approach described in Section 2.8.

**Table 1.** Treatment of antagonistic assay, *Ganoderma* **dosage** assay, and biological control.

Experiment	Treatment group	Details
Experiment 1 (Dual culture assay)	Pathogen alone	*G. boninense*
	Pathogen + *T. asperellum*	*G, boninense* + *T. asperellum*
	Pathogen + *T. virens*	*G, boninense* + *T. virens*
Experiment 2 (Dosage assay)	Healthy control	0 g pot^−1^*G. boninense*
	Dose 1	30 g pot^−1^ *G. boninense*
	Dose 2	50 g pot^−1^ *G. boninense*
	Dose 3	70 g pot^−1^ *G. boninense*
	Dose 4	150 g pot^−1^ *G. boninense*
Experiment 3 (Biocontrol Evaluation Assay)	Healthy control	Sterile medium 140 g pot^−1^ (no inoculum)
	Pathogen-only control	Sterile medium 70 g pot^−1^ + *G. boninense* 70 g pot^−1^
	*T. asperellum*	*G. boninense* 70 g pot^−1^ + *T. asperellum* 140 g pot^−1^
	*T. virens*	*G. boninense* 70 g pot^−1^ + *T. virens* 140 g pot^−1^
	**Consortium**	*G. boninense* 70 g pot^−1^ + *T. asperellum* 70 g pot^−1^ + *T. virens* 70 g pot^−1^

### 2.8. Experiment 3: greenhouse evaluation of trichoderma biocontrol against ganoderma

A greenhouse experiment was conducted to evaluate the biocontrol efficacy of *Trichoderma asperellum* (UBPg.3), *Trichoderma virens* (EXPg.2), and their consortium against *Ganoderma boninense* on oil palm seedlings. Seedlings were grown in sterilized soil medium and used at three months after planting. Colonized solid-substrate inocula of *G. boninense* and *Trichoderma* were prepared on a ground corn–sawdust carrier and used for inoculation. Treatments consisted of five groups: (i) healthy control (sterile medium, no inoculum), (ii) pathogen-only control (*G. boninense* inoculum), (iii) *T. asperellum* + *G. boninense*, (iv) *T. virens* + *G. boninense*, and (v) *Trichoderma* consortium (*T. asperellum* + *T. virens*) + *G. boninense* (see Table 1 for inoculum dosages and compositions).

The pathogen dose used in this experiment (70 g pot⁻¹) was selected based on the dose–response results of Experiment 2, which identified this level as the lowest dose that consistently and significantly induced detectable disease symptoms by 7 d after inoculation while avoiding the saturating disease pressure observed at 150 g pot⁻¹. The *Trichoderma* inoculum dose (140 g pot⁻¹), corresponding to a 2:1 antagonist-to-pathogen ratio, was chosen to ensure sufficient antagonist biomass for early rhizosphere colonization and substrate competition prior to pathogen establishment. For mixed inoculation (co-inoculation), antagonist and pathogen inocula were thoroughly mixed according to the designated dosages and incorporated into the root zone of each pot. A total of 35 seedlings were used, with seven seedlings per treatment. Each pot containing one seedling was considered one experimental replicate (experimental unit). Plants were maintained under greenhouse conditions, cared for daily, and assessed weekly for symptom development. Assessments were continued until 42 d after inoculation (DAI).

Disease severity was quantified using a leaf-based Disease Severity Index (DSI) based on leaf damage symptoms, obtained through visual assessment (chlorosis and necrosis), with the number of leaves adjusted for each plant. Observations were conducted consistently by a single researcher to minimize bias and increase data accuracy. For each seedling, all observable leaves were scored individually using a categorical rating scale. Leaf scores were summarized to generate one DSI value per pot (replicate) at each observation time. DSI was calculated as:

(2) $$\mathrm{DSI}(\%)=\frac{\sum (n\;\times \;v)}{\left(N\;\times \;Z\right)}\times 100\%$$

where *n* is the number of leaves in each disease category, *v* is the disease rating score (0, 1, 3, 5, 7), *N* is the total number of leaves assessed, and *Z* is the maximum rating score (7). Disease categories were defined based on the proportion of symptomatic leaf area as follows: 0 = healthy; 1 = 1–25% symptomatic; 3 = 26–50% symptomatic; 5 = 51–75% symptomatic; and 7 = 76–100% symptomatic.

## 3. Results

### 3.1. Morphological and molecular characterization of fungal isolates

#### 3.1.1. Ganoderma isolate UBGb.1

**Figure 1.**
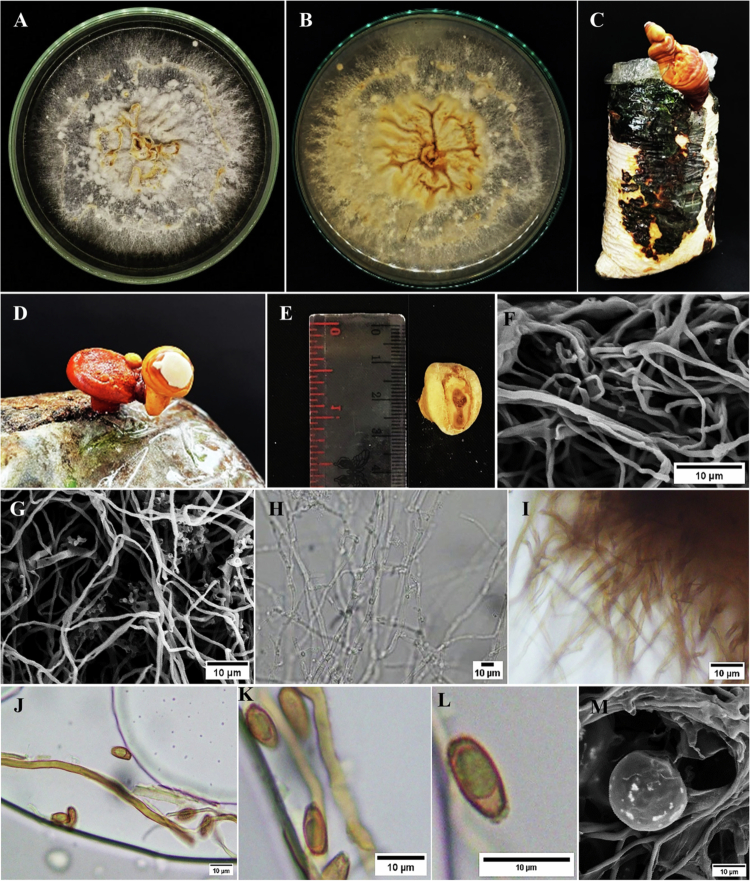
Morphological characterization of *Ganoderma* isolate UBGb.1. (A) Colony growth on PDA (obverse), (B) colony morphology on PDA (reverse), (C–E) fruiting body (basidiocarp) morphology, (F,G) SEM images of mycelia collected from PDA cultures, (H,I) hyphae observed under a light microscope, (J–L) basidiospores observed under a light microscope and associated with the fruiting body, and (M) spherical thick-walled structures observed under SEM.

The morphological characteristics of isolate UBGb.1 were examined using macroscopic and microscopic observations. On PDA, UBGb.1 initially produced a white, cottony colony that gradually developed brownish pigmentation at the colony margin and central area, with visible aerial mycelia (Figure 1A,B). A basidiocarp (fruiting body) was observed as a macroscopic reproductive structure (Figure 1C–E). Microscopically, hyaline, septate hyphae were observed under a light microscope (Figure 1H,I), while SEM revealed dense mycelial networks from PDA cultures (Figure 1F,G). Basidiospores with brownish coloration and an oval-to-elongated morphology were observed under a light microscope (Figure 1J–L), along with spherical thick-walled structures (Figure 1M).

Molecular identification based on Tef1α sequencing was used to further infer the taxonomic placement of UBGb.1. Sanger sequencing of the Tef1α amplicon yielded a consensus sequence of 807 bp. Phylogenetic analysis indicated that UBGb.1 clustered close to *G. boninense* reference sequences (e.g., accession no. MW653896.1), and **t**he closest BLASTn match showed 99.88% sequence similarity to *G. boninense* isolate MD21 (GenBank accession no. MW653896.1) (Figure 4a). The UBGb.1 Tef1α sequence generated in this study has been deposited in GenBank under accession PZ314419.1. Taken together, these results indicate that UBGb.1 belongs to the genus *Ganoderma* and is closely related to the *G. boninense* species complex.

#### 3.1.2. *Trichoderma asperellum* isolate UBPg.3

**Figure 2.**
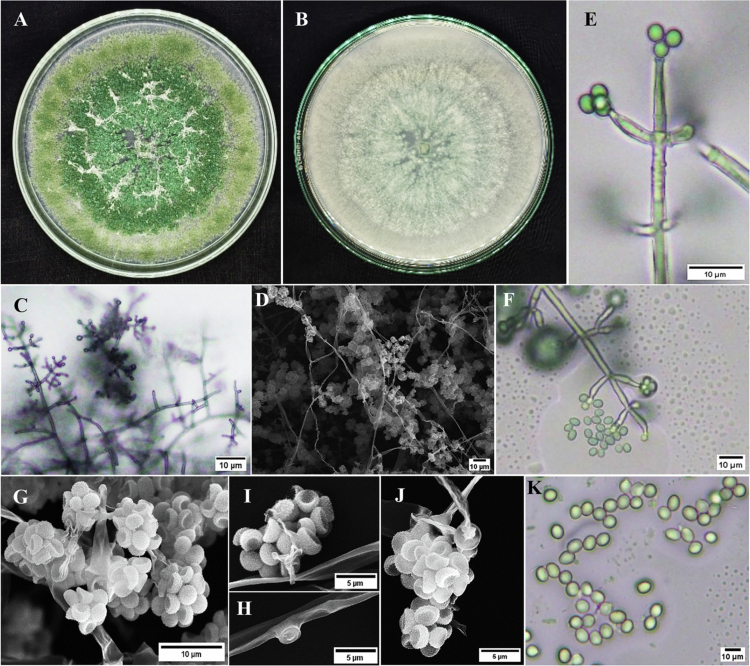
Morphological characterization of *Trichoderma asperellum* isolate UBPg.3. (A) Colony growth on PDA (obverse), (B) colony morphology on PDA (reverse), (C) hyphae observed under a light microscope, (D) SEM image of mycelia, (E) phialides, (F) conidiophores, (G) SEM image of conidiophores, (H) SEM image of chlamydospores, (I,J) SEM images of conidia, and (K) conidia observed under a light microscope.

Macroscopically, isolate UBPg.3 formed a dark-green colony with a slightly rough and dry surface, showing dense growth that uniformly covered the PDA plate (Figure 2A,B). Microscopically, septate hyphae and reproductive structures typical of *Trichoderma* were observed. Light microscopy showed septate hyphae (Figure 2C), phialides producing clustered conidia (Figure 2E), and erect, branched conidiophores (Figure 2F). SEM observations further confirmed dense mycelial structures (Figure 2D), conidiophore architecture (Figure 2G), and the presence of chlamydospores (Figure 2H). Conidia were observed both under SEM (Figure 2I,J) and under a light microscope (Figure 2K). ITS sequencing showed that UBPg.3 has a 578 bp assembly sequence, as well as phylogenetic analysis that placed UBPg.3 within the *T. asperellum* clade, showing 100% similarity to a *Trichoderma asperellum* reference sequence (accession no. MT133310.1) (GenBank accession no. PX470710.1) (Figure 4b). These results support the identification of UBPg.3 as *T. asperellum*.

#### 3.1.3. *Trichoderma virens* isolate EXPg.2

**Figure 3.**
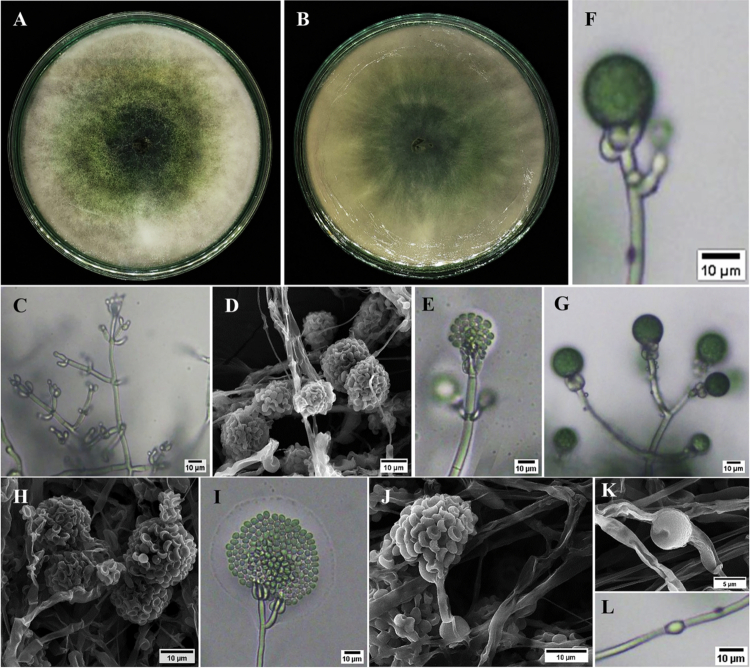
Morphological characterization of *Trichoderma virens* isolate EXPg.2. (A) Colony growth on PDA (obverse), (B) colony morphology on PDA (reverse), (C) hyphae observed under a light microscope, (D) SEM image of mycelia, (E,F) phialides, (G) conidiophores, (H) SEM image of conidiophores, (I) conidia observed under a light microscope, (J) SEM image of conidia, (K) SEM image of chlamydospores, and (L) chlamydospores observed under a light microscope.

**Figure 4.**
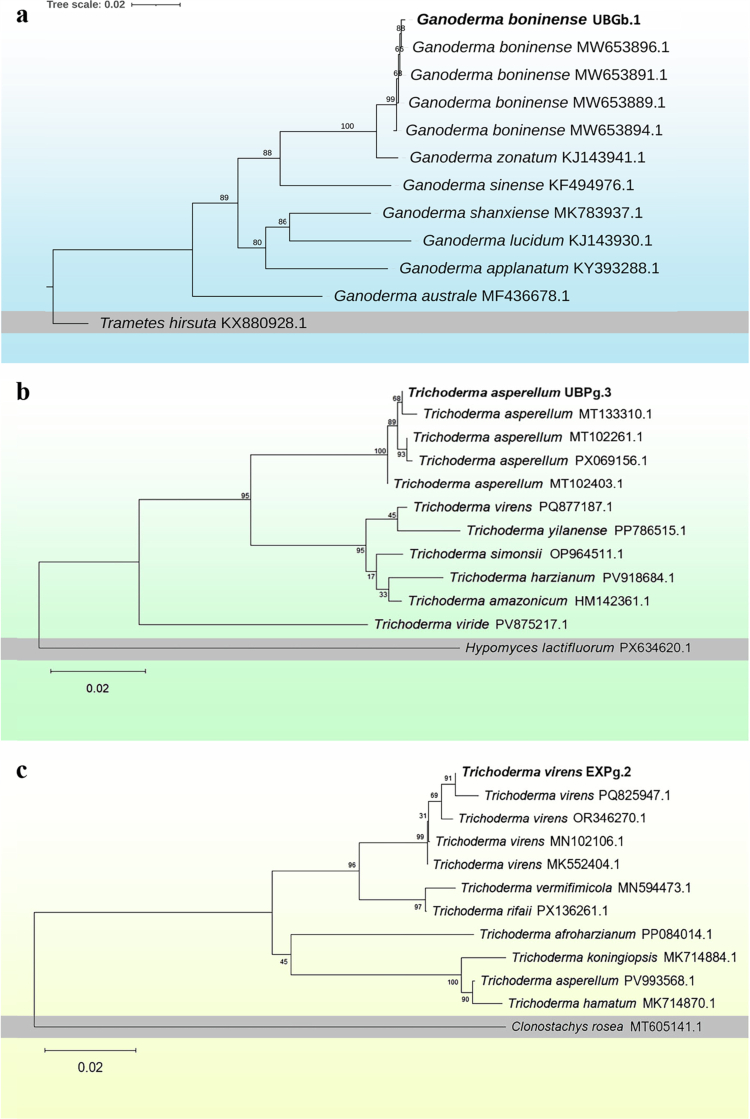
Phylogenetic placement of the study isolates based on Tef1α (panel a) and ITS rDNA (panels b,c): (a) UBGb.1 clustering with *Ganoderma boninense* reference sequences; (b) UBPg.3 within the *Trichoderma asperellum* clade; and (c) EXPg.2 within the *Trichoderma virens* clade. Node labels indicate bootstrap support values (%), and scale bars represent substitutions per site (0.02 in all panels). Trees were rooted using the outgroup taxa shown in each panel: (a) *Trametes hirsuta* KX880928.1, (b) *Hypomyces lactifluorum* PX634620.1, and (c) *Clonostachys rosea* MT605141.1. GenBank accessions of the three study isolates: UBGb.1 (PZ314419.1), UBPg.3 (PX470710.1), and EXPg.2 (PX470711.1).

Isolate EXPg.2 showed a dark-green colony center with a white margin and a smooth, cotton-like texture, with rapid growth that covered the PDA plate (Figure 3A,B). Microscopically, septate hyphae and reproductive structures typical of *Trichoderma* were observed. Light microscopy showed septate hyphae (Figure 3C), phialides (Figure 3E,F), and conidiophores (Figure 3G), while SEM confirmed mycelial structures and conidiophore architecture (Figure 3D,H). Conidia were observed under both a light microscope (Figure 3I) and SEM (Figure 3J), and chlamydospores were observed under SEM and light microscopy (Figure 3K,L). The BLASTn analysis of ITS sequences reveals an assembly sequence of 594 bp that is 100% similar to *Trichoderma virens*. Consistently, phylogenetic analysis placed EXPg.2 close to *T. virens* reference sequences (e.g., accession no. PQ825947.1), and EXPg.2 was deposited as *T. virens* EXPg.2 (accession no. PX470711.1) (Figure 4c). These findings indicate that EXPg.2 belongs to the *T. virens* species complex.

### 3.2. Experiment 1: in vitro antagonism assay against *Ganoderma boninense*

**Figure 5.**
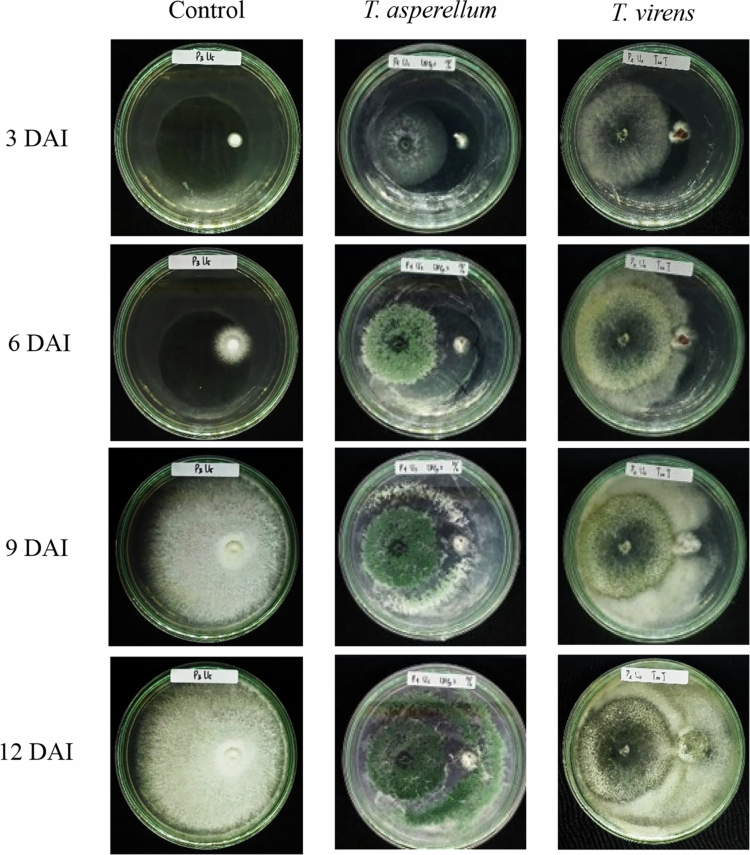
*In vitro* dual-culture antagonism of *Trichoderma asperellum* UBPg.3 and *Trichoderma virens* EXPg.2 against *Ganoderma* isolate UBGb.1 on PDA.

The *in vitro* antagonism assay (Table 2) showed that the growth of the fungus *G. boninense* was significantly (*p* < 0.05) inhibited by the addition of *T. asperellum* and *T. virens*. The diameter of the fungus in the petri dish was measured, and the results showed that the growth of *G. boninense* in the fungal treatment of both *Trichoderma* isolates decreased in diameter from 3 DAI to 12 DAI compared to the control treatment (Figure 5). For comparison, the diameter of the control treatment was 8.58 cm, while the final observation showed that the diameter of *G. boninense* treated with *T. asperellum* was 1.53 cm and that treated with *T. virens* was 1.63 cm. Based on these diameter measurements, *T. asperellum* achieved the highest inhibition of *G. boninense* mycelial growth (51.14%), while *T. virens* produced a lower but substantial inhibition (35.90%). Overall, *T. asperellum* consistently exhibited stronger antagonistic activity than *T. virens* under *in vitro* conditions, indicating greater potential as a biocontrol candidate against *G. boninense*.

**Table 2.** Percentage inhibition of *Ganoderma boninense* by *Trichoderma* isolates in dual culture.

Treatments	Inhibition against *Ganoderma boninense* (%)				
	1 DAI	3 DAI	6 DAI	9 DAI	12 DAI
Control	0.00 ± 0.00^a^	0.00 ± 0.00^a^	0.00 ± 0.00^a^	0.00 ± 0.00^a^	0.00 ± 0.00^a^
*G. boninense* + *T. asperellum*	8.33 ± 16.66^a^	46.23 ± 7.54^c^	46.83 ± 3.72^c^	51.14 ± 5.07^c^	51.14 ± 5.07^c^
*G. boninense* + *T. virens*	0.00 ± 0.00^a^	29.76 ± 11.04^b^	31.85 ± 15.07^b^	34.09 ± 15.09^b^	35.90 ± 13.13^b^

DAI = Day After Inoculation.

Values are mean ± SD (*n* = 4). Different letters within a column indicate significant differences (LSD test, *p* ≤ 0.05).

### 3.3. Experiment 2: *Ganoderma boninense* dosage assay on two-month-old oil palm seedlings

Based on the dosage assay, higher *G. boninense* inoculum levels (0, 30, 50, 70, and 150 g pot⁻¹) generally accelerated symptom development and increased disease severity in oil palm seedlings. Statistical analysis (Table 3) showed no significant differences among treatments at 7 DAI; however, early symptoms were already detected in the 70 g pot⁻¹ and 150 g pot⁻¹ treatments (DSI ≈ 9%). At 14 DAI, disease severity differed significantly among doses: the 150 g pot⁻¹ treatment produced the highest severity and was significantly higher than the other treatments, whereas the 70 g pot⁻¹ treatment showed an intermediate mean severity but was not significantly different from the lower doses at this time point. By 21 and 28 DAI, both 70 g pot⁻¹ and 150 g pot⁻¹ consistently resulted in substantially higher disease severity than the lower doses, and these two higher-dose treatments were not significantly different from each other. Visual observations (Figure 6) supported these results, where seedlings in the healthy control (0 g pot⁻¹) and the 30 g pot⁻¹ treatment remained symptomless up to 28 DAI, whereas higher inoculum doses induced earlier leaf necrosis accompanied by wilting and basal stem discoloration. Therefore, the 70 g pot⁻¹ dose was selected as a practical working inoculum level for subsequent biocontrol evaluation because it reliably induced disease symptoms without relying on the highest inoculum dose.

**Table 3.** Disease severity (%) of *Ganoderma boninense* under different inoculum doses in two-month-old oil palm seedlings.

Treatments	Disease severity (%)			
	7 DAI	14 DAI	21 DAI	28 DAI
Healthy control (0 g pot⁻¹)	0.00 ± 0.00^a^	0.00 ± 0.00^a^	0.00 ± 0.00^a^	0.00 ± 0.00^a^
*G. boninense* 30 g pot⁻¹	0.00 ± 0.00^a^	0.00 ± 0.00^a^	0.00 ± 0.00^a^	0.00 ± 0.00^a^
*G. boninense* 50 g pot⁻¹	0.00 ± 0.00^a^	2.26 ± 4.29^a^	10.12 ± 18.68^a^	14.64 ± 27.27^a^
*G. boninense* 70 g pot⁻¹	9.05 ± 18.28^a^	28.49 ± 37.92^b^	58.21 ± 41.84^b^	61.79 ± 42.04^b^
*G. boninense* 150 g pot⁻¹	9.52 ± 4.88^a^	68.45 ± 27.70^c^	82.74 ± 11.61^b^	82.74 ± 11.61^b^

DAI = Day After Inoculation.

Values represent mean±SD (*n* = 4). The significant difference (*p *≤ 0.05) is indicated by the letters using one-way ANOVA followed by LSD test.

Figure 6.Ganoderma boninense inoculation dose test on oil palm seedlings at 7, 14, 21, and 28 d after inoculation (DAI). (a) Healthy control; (b) *G. boninense* dose 30 g pot⁻¹; (c) *G. boninense* dose 50 g pot⁻¹; (d) *G. boninense* dose 70 g pot⁻¹; (e) *G. boninense* dose 150 g pot⁻¹.Composite scientific figure of potted palm-like seedlings under five experimental treatments (a–e) observed at 7, 14, 21, and 28 days after inoculation (DAI). Panels a–c show mostly healthy green seedlings with minor yellowing over time, while panel d shows moderate leaf browning and wilting. Panel e shows severe disease progression, with leaves turning yellow-orange to brown and becoming necrotic by 21–28 DAI.

**Figure:**
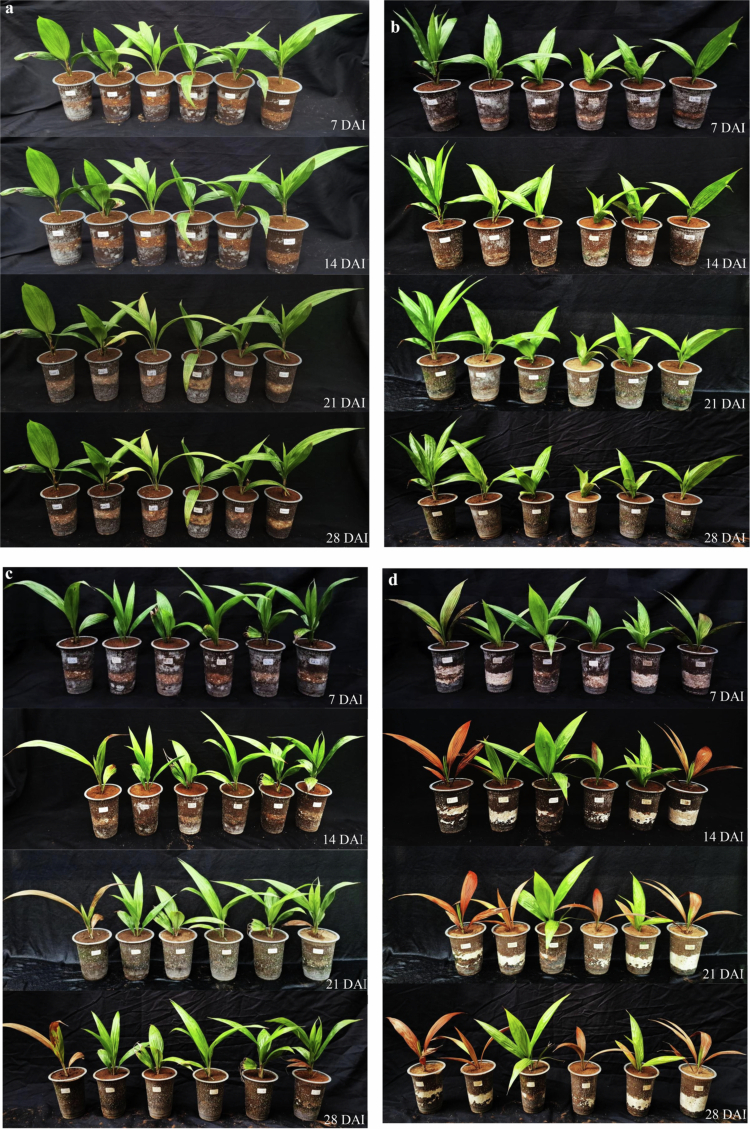

**Figure:**
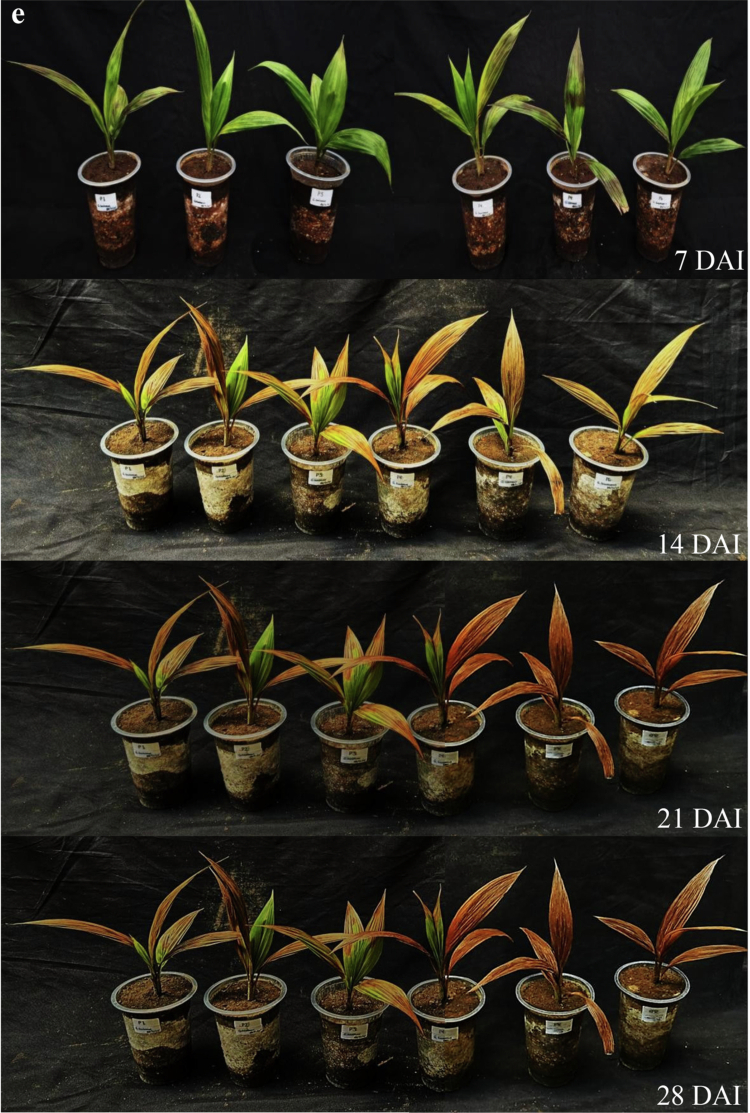

### 3.4. Experiment 3: biological control of *Ganoderma boninense* using *Trichoderma asperellum* and *Trichoderma virens*

**Figure 7.**
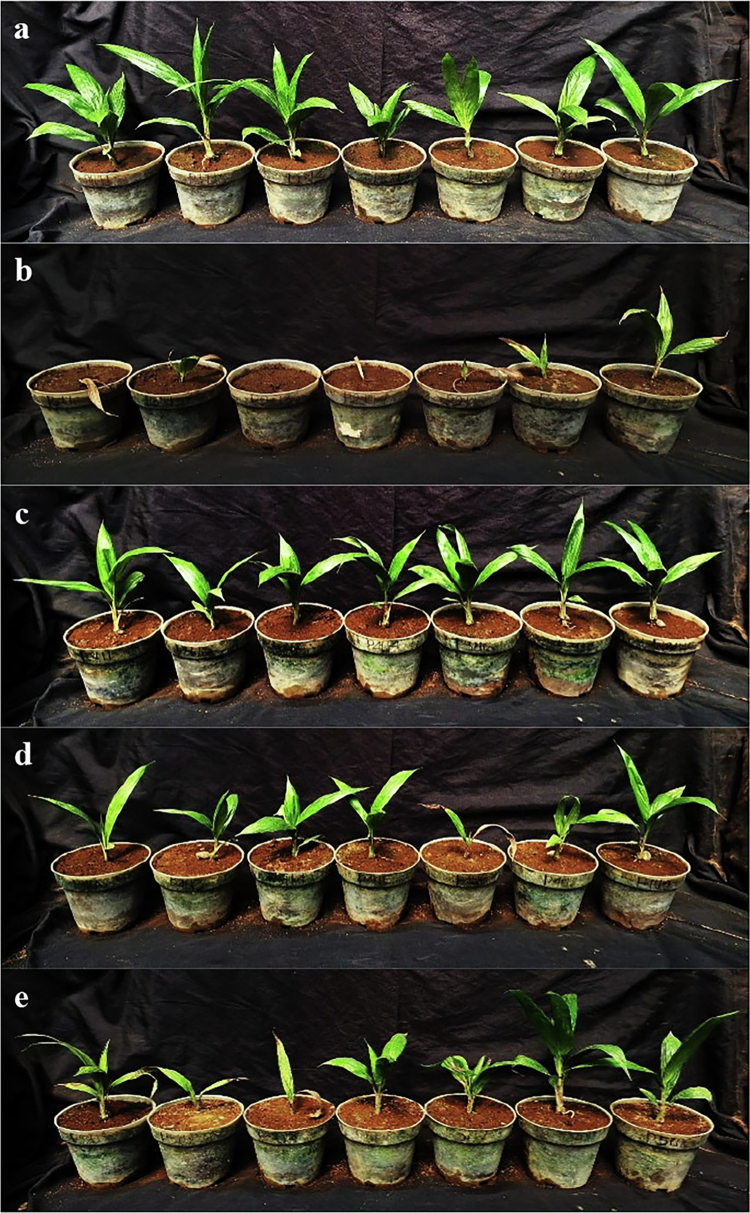
Effectiveness of Antagonist Fungi Against *Ganoderma boninense* on Oil Palm Seedlings (a) Healthy control; (b) Pathogen-only control; (c) *T. asperellum* 140 g pot⁻¹ + *G. boninense* 70 g pot⁻¹; (d) *T. virens* 140 g pot⁻¹ + *G. boninense* 70 g pot⁻¹; (e) Consortium 140 g pot⁻¹ (*T. asperellum* 70 g pot⁻¹ + *T. virens* 70 g pot⁻¹) + *G. boninense* 70 g pot⁻¹.

Based on greenhouse observations (Table 4), *Trichoderma* treatments effectively suppressed disease development caused by *Ganoderma boninense* compared with the pathogen-only control (Figure 7). Application of *T. asperellum* solid-substrate (corn–sawdust) inoculum (140 g pot⁻¹) applied by mixed inoculation with *G. boninense* inoculum (70 g pot⁻¹) maintained a DSI of 0.00% at all observation times up to 42 DAI, with no visible symptoms observed throughout the experiment. In contrast, the pathogen-only control (70 g pot⁻¹ *G. boninense*) exhibited progressive disease development, reaching 83.67% DSI at 42 DAI. Treatments with *T. virens* (140 g pot⁻¹) + *G. boninense* (70 g pot⁻¹) and the *Trichoderma* consortium + *G. boninense* (70 g pot⁻¹) also resulted in low disease levels, with DSI values of 6.80% and 10.88% at 42 DAI, respectively. Overall, these results support the biocontrol potential of *Trichoderma* spp. against *G. boninense* under *in vivo* conditions, with *T. asperellum* showing the strongest suppression in this study.

**Table 4.** Biocontrol of *Ganoderma boninense* in oil palm seedlings using *Trichoderma* isolates.

Treatments	Disease severity (%)					
	7 DAI	14 DAI	21 DAI	28 DAI	35 DAI	42 DAI
Healthy control (non-inoculated)	0.00 ± 0.00^a^	0.00 ± 0.00^a^	0.00 ± 0.00^a^	0.00 ± 0.00^a^	0.00 ± 0.00^a^	0.00 ± 0.00^a^
Pathogen-only control (*G. boninense* 70 g pot⁻¹)	9.18 ± 12.33^b^	28.17 ± 38.93^b^	51.70 ± 38.63^b^	61.90 ± 33.33^b^	75.51 ± 31.99^b^	83.67 ± 22.47^b^
*T. asperellum* 140 g pot⁻¹ + *G. boninense* 70 g pot⁻¹	0.00 ± 0.00^a^	0.00 ± 0.00^a^	0.00 ± 0.00^a^	0.00 ± 0.00^a^	0.00 ± 0.00^a^	0.00 ± 0.00^a^
*T. virens* 140 g pot⁻¹ + *G. boninense* 70 g pot⁻¹	2.04 ± 5.39^a^	5.44 ± 14.39^a^	6.80 ± 17.99^a^	6.80 ± 17.99^a^	6.80 ± 17.99^a^	6.80 ± 17.99^a^
**Consortium** 140 g pot⁻¹ + *G. boninense* 70 g pot⁻¹	0.68 ± 2.54^a^	2.72 ± 2.32^a^	4.08 ± 4.28^a^	5.44 ± 5.78^a^	8.16 ± 10.54^a^	10.88 ± 14.21^a^

DAI = Day After Inoculation.

Values represent mean ± SD (*n* = 7). The significant difference (*p* ≤ 0.05) is indicated by the letters using one-way ANOVA followed by LSD test.

## 4. Discussion

### 4.1. Identification and relevance to biocontrol

Morphological and molecular characterization of fungal isolates provide a crucial basis for interpreting the antagonistic effectiveness of *Trichoderma* against *Ganoderma* associated with basal stem rot in oil palm. Isolate UBGb.1 showed colony and microscopic features consistent with the genus *Ganoderma*, including cottony white colonies that gradually turned brown and hyaline septate hyphae, together with basidiospores observed on the basidiocarp surface. Tef1α sequence analysis placed UBGb.1 within *Ganoderma* and closest to *G. boninense***–**related references (BLASTn similarity 99.88%), confirming species-level placement within the *G. boninense* species complex at a similarity level that meets the conventional threshold for species delineation. This supports the view that integrating morphological traits with molecular markers improves confidence in identifying plant-associated fungi.^6^ In contrast, antagonist isolates UBPg.3 and EXPg.2 were identified as *Trichoderma asperellum* and *Trichoderma virens* with 100% ITS sequence identity, well above the 99.61% threshold proposed for ITS-based species delineation in filamentous fungi.^6^ Both species have been widely reported as potent biocontrol agents against soil-borne pathogens through mycoparasitism and competition for space and nutrients, as well as antibiosis mediated by hydrolytic enzymes and secondary metabolites.^7-9^

### 4.2. *In vitro* antagonism and possible contributing mechanisms

In vitro dual-culture assays showed that *T. asperellum* inhibited *G. boninense* more strongly than *T. virens* (51.14% vs. 35.90% inhibition at 12 d after incubation). This pattern is in line with reports describing strong antagonism of *T. asperellum* against *G. boninense* in oil palm, including high PIRG values in dual culture and reported hydrolytic-enzyme-related traits.^10^ The antifungal capacity of *Trichoderma* has also been associated with inhibitory volatile compounds.^11^ Some studies suggest that volatile production may increase under certain stress conditions.^12^ In addition, secondary metabolites produced by *Trichoderma* can contribute to pathogen suppression and may modulate plant–microbe interactions, potentially enhancing plant defense responses^9^; however, metabolite profiling was not conducted in the present study, and these mechanisms should be interpreted cautiously.

### 4.3. *In vivo* biocontrol performance and consortium outcome

In vivo a mixed-inoculation (co-inoculation) assay using solid-substrate (corn–sawdust) inocula showed that *T. asperellum* (140 g pot⁻¹) suppressed BSR development when mixed-inoculated with *G. boninense* (70 g pot⁻¹), resulting in 0% DSI up to 42 DAI (i.e., no visible symptoms during the observation period), whereas the pathogen-only control showed progressive disease development. Notably, the stronger antagonism of *T. asperellum* observed in dual-culture assays was consistent with its superior disease suppression in the nursery experiment. Treatments using *T. virens* or the *Trichoderma* consortium also resulted in low disease severity at 42 DAI (6.80% and 10.88%, respectively). However, under the conditions tested, the consortium did not outperform the best single-strain treatment (*T. asperellum*). Similar outcomes were reported by,^13^) where a combined treatment of *T. asperellum* and *T. virens* did not outperform a single *Trichoderma* strain in suppressing *G. boninense*. Although the underlying basis of this difference cannot be inferred from the present data, possible contributing factors—such as inter-strain interactions, niche overlap, and substrate-dependent metabolite production—are recognized in the literature for *Trichoderma*-based biocontrol systems^7^^,^^14^ and were not directly assessed in this study; targeted experiments addressing these mechanisms are outlined in the Limitations and Future Directions section below.

### 4.4. Implications

This study indicates that biocontrol effectiveness is strongly influenced by isolate-specific performance and the stability of antagonistic activity under specific host and environmental conditions. Therefore, early detection of infection is critical because it can provide a practical window for intervention before severe symptoms develop, increasing the chance of disease suppression and seedling survival, as well as improving the quality of seedlings established in the field.^15^ Beyond the scope of this work, *Trichoderma*-based biocontrol could be strengthened by integrating early, non-destructive screening tools (e.g., spectral indices and image-based phenotyping) with data-driven classification approaches to support nursery decision-making. Future research should also explore microbiome-informed strategies, including the design of *Trichoderma*-centered synthetic communities (SynComs) to shift the rhizosphere toward a disease-suppressive state (through niche competition, functional redundancy, and immune priming), which may help limit *Ganoderma* colonization and slow disease progression. Further studies should test intervention timing across disease-severity stages and validate outcomes from nursery to field conditions using survival, growth performance, and pathogen colonization indicators.

### 4.5. Limitations and future directions

Several methodological constraints of the present study should be acknowledged. (i) Although Tef1α sequencing places UBGb.1 within the *G. boninense* complex with high similarity (99.88%), multi-gene phylogenetic analysis is recommended in future work to further consolidate species-level identification. (ii) The *in vitro* confrontation used a fixed-plug format; gradient dose–response assays and direct strain–strain interaction tests were not performed and would clarify the basis of the reduced efficacy of the two-strain consortium relative to *T. asperellum* applied alone. (iii) The greenhouse trial employed a single-factor design with fixed pathogen (70 g pot⁻¹) and antagonist (140 g pot⁻¹) doses; a two-factor design varying pathogen pressure and antagonist-to-pathogen ratio, including alternative consortium ratios, is recommended to more comprehensively map biocontrol efficacy under variable inoculum pressure. (iv) Only a solid (corn–sawdust) carrier formulation was evaluated; comparative testing with liquid formulations, which differ in penetration, distribution, and stability in the rhizosphere, would strengthen translational guidance for field application. (v) Pre-trial morphometric baselines of seedlings were not systematically recorded, and disease severity scoring was performed by a single trained observer to maintain consistency rather than under blinded conditions; future trials should include pre-treatment growth data and blinded multi-rater scoring to further reduce assessment bias. Finally, although the antagonism observed here is consistent with hydrolytic-enzyme- and metabolite-mediated mechanisms—including antibiosis previously demonstrated for *T. virens* via FTIR-based metabolite profiling in our previous work^13^—direct molecular characterization of these mechanisms in the present strains was beyond the scope of this study and should be addressed in future work through comparative metabolomic and transcriptomic profiling, together with mycoparasitism imaging assays (e.g., hyphal coiling and lytic enzyme localization).

## Data Availability

The data supporting the findings of this study are available within the article. Nucleotide sequences have been deposited in the NCBI GenBank database under accession numbers PZ314419.1 (UBGb.1, Tef1α), PX470710.1 (UBPg.3, ITS), and PX470711.1 (EXPg.2, ITS). Additional raw data, including *in vitro* colony measurements, dose–response disease severity scoring, and greenhouse observation records, are available from the corresponding author upon reasonable request for academic verification, replication, and educational purposes.
